# SLC25A5 Suppresses Colorectal Cancer Growth and Metastasis Through Regulation of the EIF3A/PI3K/AKT Axis

**DOI:** 10.3390/ijms27104334

**Published:** 2026-05-13

**Authors:** Ke Ying, Xiang Zhao, Zhuo Wu, Chi Huang, Qian Wu, Zhongchen Liu

**Affiliations:** School of Medicine, Tongji University, Shanghai 200070, China; yingke159357@163.com (K.Y.); 2332316@tongji.edu.cn (X.Z.); 2511275@tongji.edu.cn (Z.W.); 13665546929@163.com (C.H.); wqian0405@163.com (Q.W.)

**Keywords:** colorectal cancer, SLC25A5, EIF3A, PI3K-AKT pathway, metastasis

## Abstract

Colorectal cancer (CRC) progression is driven by dysregulated signaling networks that promote proliferation and metastasis. While SLC25A5 is a well-characterized mitochondrial ADP/ATP transporter, its potential non-canonical roles in cancer remain unclear. This study investigated whether SLC25A5 exerts tumor-suppressive functions in CRC. Using transcriptomic datasets and clinical cohorts, we found that SLC25A5 is significantly downregulated in CRC tissues, and low expression is associated with poor patient survival. Restoration of SLC25A5 suppressed CRC cell proliferation, epithelial–mesenchymal transition (EMT), and metastasis in vitro and in vivo. Mechanistically, co-immunoprecipitation and protein stability assays suggested an association between SLC25A5 and EIF3A and indicated that SLC25A5 may promote EIF3A destabilization through the ubiquitin–proteasome pathway without altering its mRNA levels. Subcellular fractionation further suggested the presence of a cytoplasmic pool of SLC25A5, providing a potential basis for this interaction. Rescue experiments showed that EIF3A overexpression partially reversed the tumor-suppressive effects of SLC25A5. In addition, SLC25A5 expression was associated with reduced PI3K/AKT signaling activity, and pharmacological activation of AKT partially restored invasive phenotypes. Collectively, these findings suggest an SLC25A5–EIF3A–PI3K/AKT regulatory axis and reveal a potential non-canonical role for this mitochondrial carrier in tumor progression. This study provides insight into how mitochondrial proteins may influence cytoplasmic signaling pathways in cancer.

## 1. Introduction

Colorectal cancer remains a major cause of cancer-related mortality worldwide, largely due to its high metastatic potential and resistance to apoptosis [1,2]. Although advances in surgical techniques and targeted therapies have improved patient management, the molecular mechanisms that sustain CRC progression and epithelial–mesenchymal transition are still incompletely understood [3,4]. A deeper elucidation of these regulatory networks is urgently needed to identify more effective prognostic markers and therapeutic targets.

Members of the Solute Carrier Family 25 (SLC25), particularly the mitochondrial adenine nucleotide translocators (ANTs), have traditionally been characterized as essential mediators of ADP/ATP exchange across the inner mitochondrial membrane [5,6]. However, accumulating evidence suggests that certain SLC25 proteins exert context-dependent roles in tumor biology that extend beyond metabolic regulation [7,8,9,10]. SLC25A5 (ANT2), in particular, has been implicated in cancer-associated metabolic reprogramming [11], yet its function in colorectal cancer remains ambiguous. Notably, whether SLC25A5 possesses non-canonical, metabolism-independent activities—especially within the cytoplasmic compartment—has not been clearly defined. This gap raises the possibility that SLC25A5 may participate in tumor regulation through mechanisms unrelated to mitochondrial bioenergetics.

Aberrant control of protein synthesis and stability represents a central hallmark of malignant progression [12]. Eukaryotic Translation Initiation Factor 3 Subunit A (EIF3A), the largest subunit of the EIF3 complex, has emerged as a critical oncogenic driver that enhances the translation of mRNAs governing cell cycle progression, survival, and metastasis [13,14,15]. Elevated EIF3A expression has been reported in multiple aggressive cancers, including CRC [16,17,18]. Moreover, EIF3A has been suggested to function as a signaling integrator linking translational control to oncogenic pathways such as PI3K/AKT [19], a pathway frequently hyperactivated in CRC to promote proliferation and metastatic dissemination. Despite its oncogenic relevance, the upstream regulatory mechanisms that dictate EIF3A protein stability and activity in CRC remain largely undefined.

In the present study, we combined bioinformatic analyses, clinical validation, and in vitro and in vivo functional assays to investigate the role of SLC25A5 in CRC. We provide evidence that SLC25A5 suppresses CRC growth and metastasis and is associated with the regulation of EIF3A protein stability and PI3K/AKT signaling. Our findings suggest a previously uncharacterized SLC25A5/EIF3A/PI3K–AKT regulatory axis and provide new insight into the non-canonical functions of mitochondrial carrier proteins in tumor biology. These results further indicate that SLC25A5 may represent a potential therapeutic target in metastatic colorectal cancer.

## 2. Results

### 2.1. SLC25A5 Expression Is Downregulated in CRC and Correlates with Poor Prognosis

**Figure 1:**
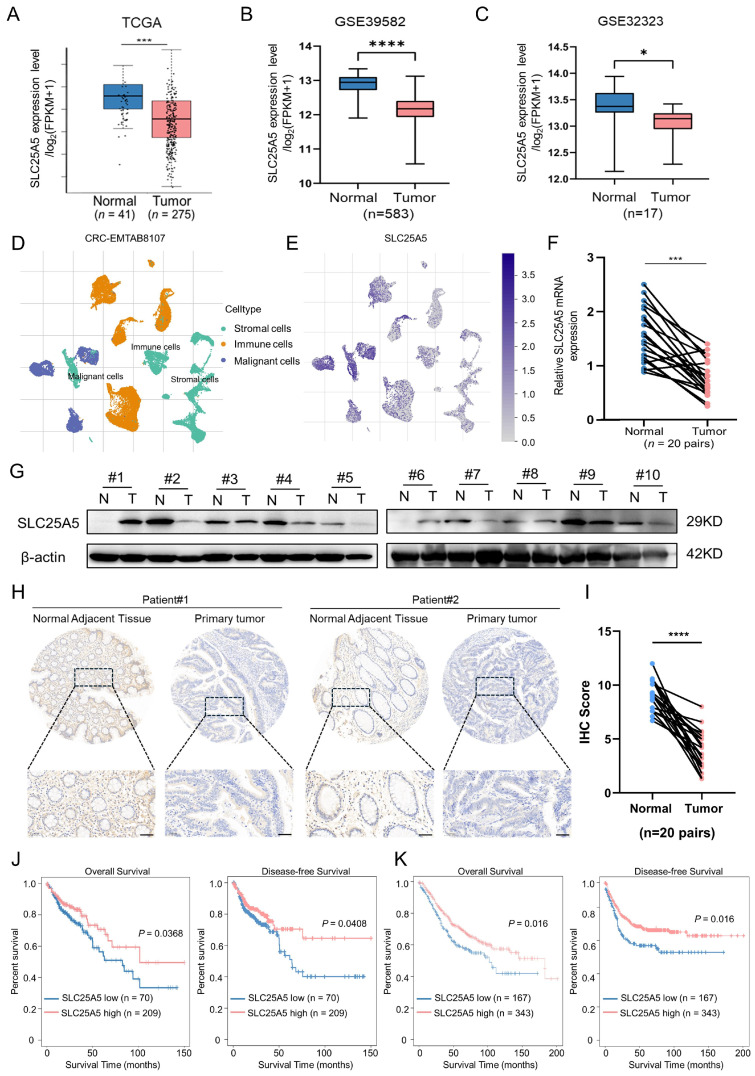
SLC25A5 expression is downregulated in CRC and correlates with poor prognosis: (**A**–**C**) Bioinformatic analysis of *SLC25A5* mRNA expression levels in colorectal cancer (CRC) tumor tissues compared to adjacent normal tissues using the TCGA cohort (**A**), GSE39582 dataset (**B**), and GSE32323 dataset (**C**). (**D**,**E**) Single-cell RNA sequencing (scRNA-seq) analysis using the CRC-EMTAB8107 dataset. (**D**) UMAP visualization categorizing the major cell types into stromal, immune, and malignant cell populations. (**E**) UMAP feature plot illustrating the expression distribution of *SLC25A5* across the different cell clusters. (**F**) Quantitative PCR (RT-qPCR) analysis of relative *SLC25A5* mRNA expression in 20 pairs of clinical CRC tissues and matched normal adjacent tissues. (**G**) Western blot images showing SLC25A5 protein levels in 10 paired CRC tumor (T) and matched normal (N) tissues (n = 10 pairs). (**H**) Representative immunohistochemistry (IHC) staining images of primary tumor sections and matched normal adjacent tissues. Enlarged views of the boxed regions are shown in the lower panels (Scale bars: 100 μm). (**I**) Statistical comparison of the IHC scores between normal and tumor tissues in 20 patient pairs. The IHC score was determined by multiplying the staining intensity score by the percentage of positively stained cells. (**J**,**K**) Kaplan–Meier survival curves evaluating the prognostic value of SLC25A5. Overall survival (OS) and disease-free survival (DFS) analyses based on SLC25A5 expression levels in the TCGA-COAD cohort (**J**) and the GSE39582 cohort (**K**). * *p* < 0.05, *** *p* < 0.001, **** *p* < 0.0001, assessed by paired Student’s *t*-test or log-rank test.

To evaluate the differential expression of SLC25A5 in colorectal cancer, we initially characterized its expression profile using public datasets. Bioinformatic analysis of the TCGA cohort (Figure 1A) and GSE datasets (GSE39582, Figure 1B; GSE32323, Figure 1C) consistently revealed that SLC25A5 mRNA levels were significantly downregulated in tumor tissues compared to adjacent normal tissues. To further define the cellular context of SLC25A5 expression within the tumor microenvironment, we analyzed single-cell RNA sequencing data from the CRC-EMTAB8107 dataset. UMAP visualization classified cells into stromal, immune, and malignant populations (Figure 1D), revealing that SLC25A5 was broadly expressed across multiple cell types but exhibited relatively enriched expression within malignant epithelial cells (Figure 1E).

These findings were further validated in our local clinical cohort. Quantitative PCR analysis of 20 pairs of CRC tissues and matched normal adjacent tissues confirmed a consistent reduction in SLC25A5 mRNA in tumor samples (Figure 1F). At the protein level, Western blot analysis of 10 patient pairs corroborated this trend (Figure 1G). Furthermore, immunohistochemistry staining of primary tumor sections and matched normal tissues provided spatial validation of SLC25A5 expression patterns (Figure 1H); strong SLC25A5 immunoreactivity was observed in normal glandular structures, whereas staining was significantly attenuated in cancerous regions. This downregulation was further supported by quantitative IHC scoring across 20 paired clinical samples (Figure 1I).

Finally, to evaluate the prognostic value of SLC25A5 in CRC, we performed Kaplan–Meier survival analyses across independent patient cohorts. Data from both the TCGA-COAD database and the GEO dataset GSE39582 yielded concordant results, showing that patients with higher SLC25A5 expression had significantly improved overall survival and disease-free survival compared to those with low expression (Figure 1J,K). This cross-cohort consistency supports SLC25A5 as a potential favorable prognostic indicator in CRC. Collectively, these findings suggest that SLC25A5 is frequently downregulated in CRC and is associated with poor clinical outcomes.

### 2.2. SLC25A5 Inhibits CRC Progression by Suppressing Proliferation and Promoting Apoptosis

**Figure 2:**
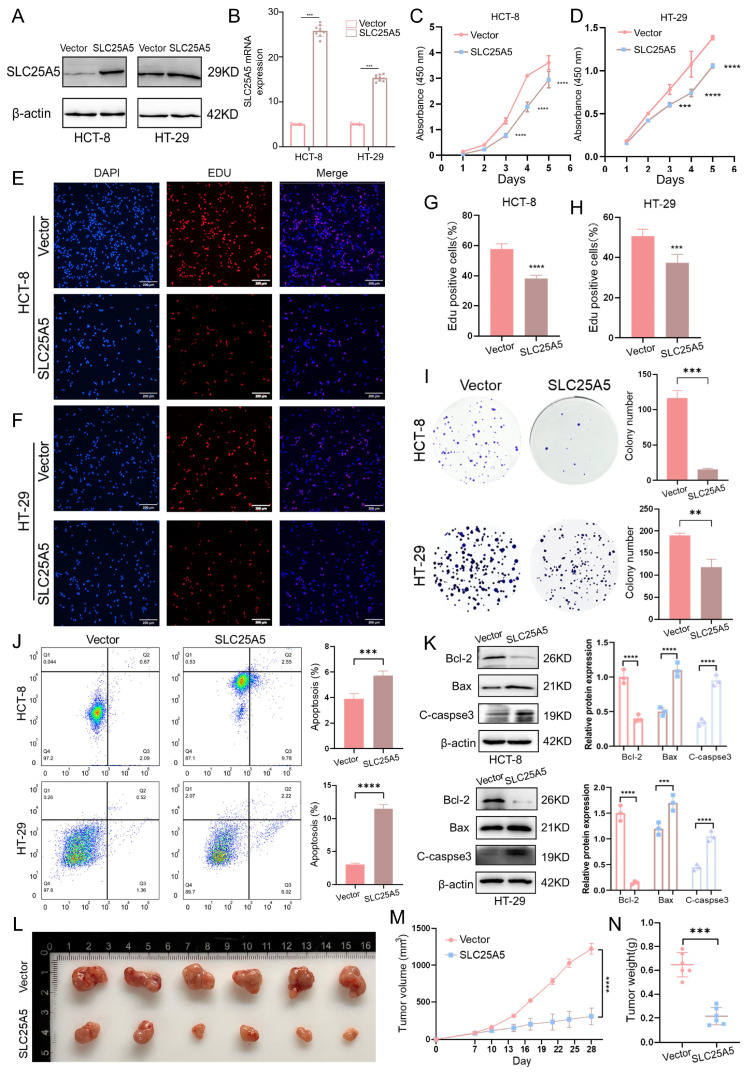
SLC25A5 inhibits CRC progression by suppressing proliferation and promoting apoptosis: (**A**,**B**) Western blot (**A**) and RT-qPCR (**B**) analysis confirming the successful overexpression of SLC25A5 in HCT-8 and HT-29 cell lines. (**C**,**D**) CCK-8 proliferation assays showing the growth rates of HCT-8 (**C**) and HT-29 (**D**) cells transfected with either empty vector or SLC25A5 expression plasmid. (**E**–**H**) Representative images (**E**,**F**) and quantitative analysis (**G**,**H**) of EdU incorporation assays in HCT-8 and HT-29 cells (Nuclei were stained with DAPI (blue), and proliferating cells were stained with EdU (red). Scale bars: 200 μm). (**I**) Representative images and statistical analysis of colony formation assays. (**J**) Flow cytometry analysis and quantification of apoptotic cells in the indicated groups. (**K**) Western blot analysis of Bcl-2, Bax, and Cleaved-caspase3 (C-caspase3) protein levels in HCT-8 and HT-29 cells. (**L**–**N**) Representative images of xenograft tumors (**L**), tumor growth curves (**M**), and final tumor weights (**N**) from nude mice injected with the indicated HCT-8 cells (n = 6 per group). ** *p* < 0.01, *** *p* < 0.001, **** *p* < 0.0001.

To investigate the functional role of SLC25A5 in colorectal cancer progression, we first assessed its endogenous expression across a panel of CRC cell lines. Consistent with our clinical analyses, SLC25A5 expression was relatively low in most CRC cell lines compared to the normal colonic epithelial cell line FHC (Supplementary Figure S1). Based on this expression pattern, HCT-8 and HT-29 cells, which exhibited the lowest SLC25A5 expression, were selected for subsequent restoration-of-function studies. We then established stable SLC25A5-overexpressing HCT-8 and HT-29 cell lines, and successful overexpression was confirmed at both the protein (Figure 2A) and mRNA levels (Figure 2B). A series of in vitro and in vivo assays were subsequently performed using these models.

First, to assess the effect of SLC25A5 on CRC cell proliferation, we performed CCK-8, EdU incorporation, and colony formation assays. CCK-8 assays demonstrated that forced expression of SLC25A5 significantly attenuated the proliferation of both HCT-8 (Figure 2C) and HT-29 (Figure 2D) cells compared to vector controls. Consistent with these findings, EdU incorporation assays showed a marked reduction in the proportion of DNA-synthesizing cells in both cell lines (Figure 2E–H). Furthermore, colony formation assays revealed a substantial decrease in colony numbers (Figure 2I), indicating that SLC25A5 suppresses the proliferative and clonogenic capacity of CRC cells.

We next examined whether SLC25A5 affects cell survival. Flow cytometric analysis showed a significant increase in apoptotic cells in SLC25A5-overexpressing HCT-8 and HT-29 groups compared to controls (Figure 2J). Consistently, Western blot analysis demonstrated that SLC25A5 overexpression altered the expression of apoptosis-related proteins, characterized by decreased Bcl-2 levels and increased Bax and cleaved caspase-3 levels (Figure 2K).

The tumor-suppressive effect of SLC25A5 was further evaluated in vivo using a nude mouse xenograft model (Figure 2L). Mice injected with SLC25A5-overexpressing HCT-8 cells exhibited significantly reduced tumor growth, as reflected by decreased tumor volume (Figure 2M) and final tumor weight (Figure 2N) over a 28-day period compared to the control group. Taken together, these findings support a tumor-suppressive role for SLC25A5 in CRC, associated with inhibition of cell proliferation and induction of apoptosis.

### 2.3. SLC25A5 Inhibits CRC Metastasis by Suppressing EMT and Cell Motility

**Figure 3:**
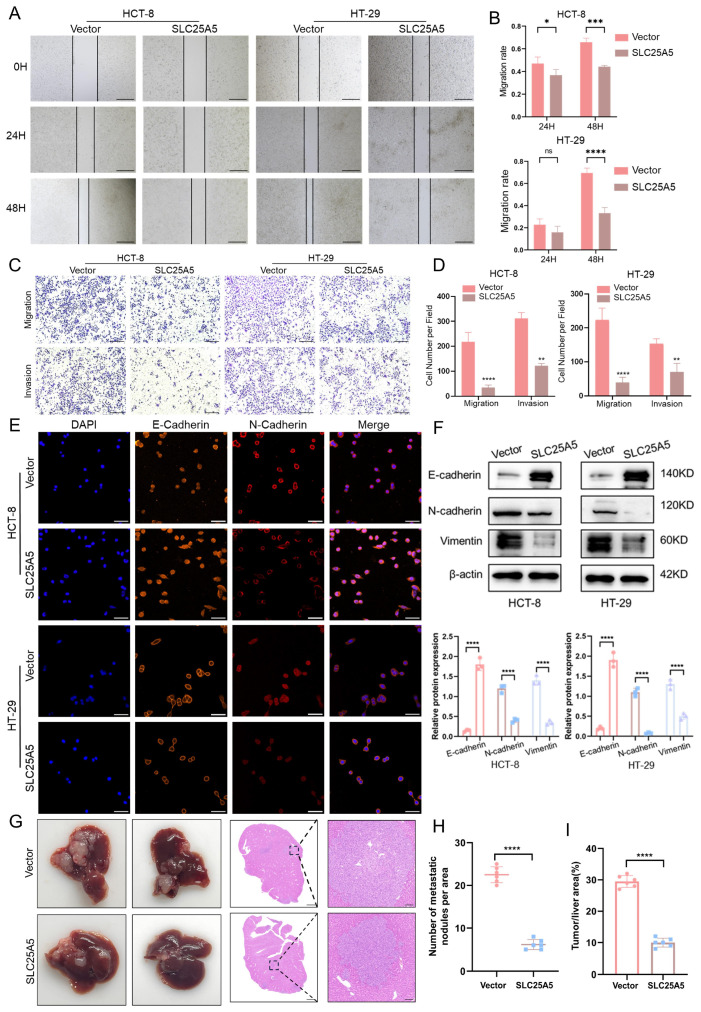
SLC25A5 inhibits CRC metastasis by suppressing EMT and cell motility: (**A**,**B**) Representative images (**A**) and statistical analysis (**B**) of wound healing assays in HCT-8 and HT-29 cells at 0, 24, and 48 h (Scale bars: 500 μm). (**C**,**D**) Representative images (**C**) and quantitative analysis (**D**) of Transwell migration and Matrigel invasion assays (Scale bars: 100 μm). (**E**) Immunofluorescence staining of E-cadherin (orange) and N-cadherin (red) in the indicated CRC cells; nuclei were stained with DAPI (blue) (Scale bars: 50 μm). (**F**) Western blot analysis of E-cadherin, N-cadherin, and Vimentin protein levels in HCT-8 and HT-29 cells. (**G**) Representative images of livers and H&E-stained liver sections from the in vivo metastasis model. (**H**,**I**) Quantification of the number of metastatic nodules per area (**H**) and the percentage of tumor/liver area (**I**). * *p* < 0.05, ** *p* < 0.01, *** *p* < 0.001, **** *p* < 0.0001.

To assess the effect of SLC25A5 on CRC cell motility, we performed wound healing, Transwell migration, and Matrigel invasion assays. Wound healing assays showed that overexpression of SLC25A5 significantly delayed scratch closure in both HCT-8 and HT-29 cells at 24 and 48 h compared to vector controls (Figure 3A,B), indicating reduced migratory capacity. This finding was further supported by Transwell migration and Matrigel invasion assays, in which SLC25A5-overexpressing cells exhibited a marked decrease in the number of cells migrating through the membrane or invading through the matrix (Figure 3C,D).

Consistent with these phenotypic changes, immunofluorescence (Figure 3E) and Western blot analysis (Figure 3F) demonstrated that SLC25A5 modulates the expression of epithelial–mesenchymal transition (EMT)-related markers. Specifically, the epithelial marker E-cadherin was upregulated, whereas the mesenchymal markers N-cadherin and vimentin were downregulated in SLC25A5-overexpressing HCT-8 and HT-29 cells.

The anti-metastatic effect of SLC25A5 was further evaluated using an in vivo liver metastasis model. Mice injected with SLC25A5-overexpressing cells developed significantly fewer and smaller metastatic nodules in the liver compared to control mice (Figure 3G). Histological analysis using H&E staining further confirmed a reduction in both the number of metastatic nodules per area (Figure 3H) and the tumor-to-liver area ratio (Figure 3I). Taken together, these findings suggest that SLC25A5 suppresses CRC cell migration, invasion, and metastasis, potentially through modulation of EMT.

### 2.4. SLC25A5 Modulates the Proteasomal Degradation of EIF3A

**Figure 4:**
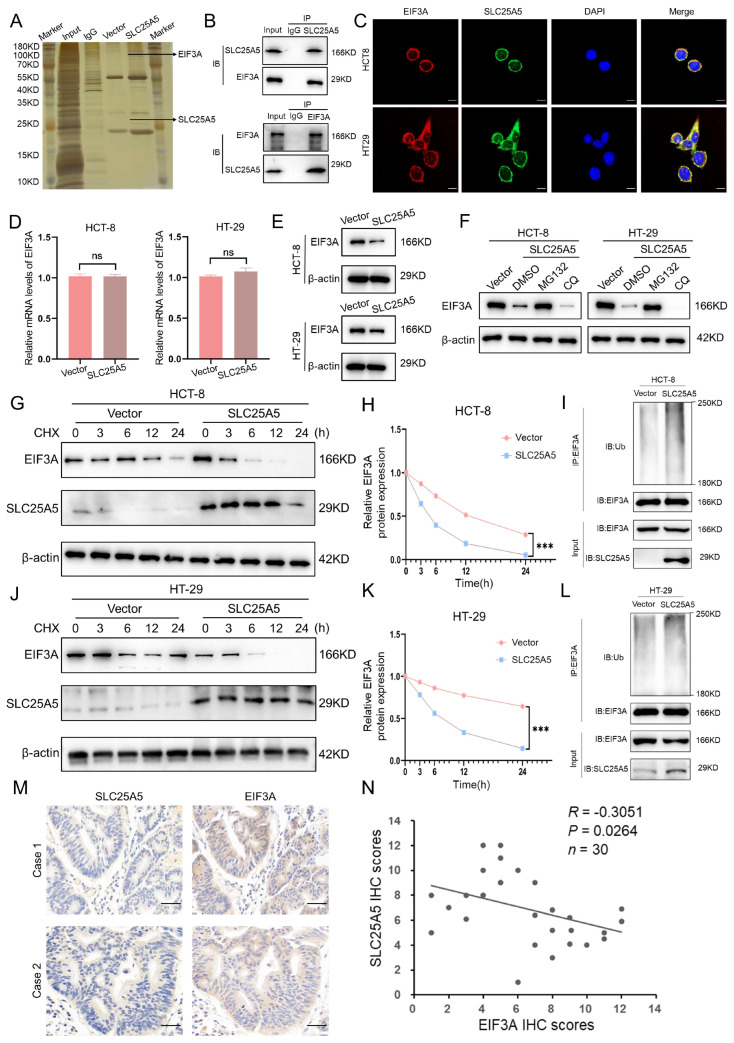
SLC25A5 Modulates the Proteasomal Degradation of EIF3A: (**A**) Silver staining showing protein bands pulled down by SLC25A5 in HCT-8 cells; arrows indicate EIF3A and SLC25A5. (**B**) Endogenous Co-IP analysis demonstrating the interaction between SLC25A5 and EIF3A in CRC cells. (**C**) Immunofluorescence images showing the co-localization of EIF3A (red) and SLC25A5 (green) in HCT-8 and HT-29 cells (Nuclei were counterstained with DAPI (blue). The yellow color in the merged images indicates the co-localization of EIF3A and SLC25A5 proteins. Scale bars: 10 μm). (**D**,**E**) RT-qPCR (**D**) and Western blot (**E**) analysis of EIF3A expression in CRC cells overexpressing SLC25A5. (**F**) Western blot analysis of EIF3A in SLC25A5-overexpressing cells treated with MG132 (10 μM) or CQ (50 μM). (**G**,**H**) Representative Western blot (**G**) and quantitative degradation curves (**H**) of EIF3A in HCT-8 cells treated with CHX (100 μg/mL) over a 24-h time course. (**I**) Ubiquitination assay showing the effect of SLC25A5 overexpression on the poly-ubiquitination of EIF3A in HCT-8 cells. (**J**,**K**) Representative Western blot (**J**) and quantitative degradation curves (**K**) of EIF3A in HT-29 cells treated with CHX (100 μg/mL). (**L**) Ubiquitination assay showing SLC25A5-mediated EIF3A poly-ubiquitination in HT-29 cells. (**M**) Representative IHC images showing the expression of SLC25A5 and EIF3A in two clinical CRC cases (Scale bars: 100 μm). (**N**) Pearson correlation analysis between SLC25A5 and EIF3A IHC scores in CRC specimens (n = 30). *** *p* < 0.001, ns, non-significant.

To identify potential interacting partners of SLC25A5, we performed co-immunoprecipitation followed by mass spectrometry (IP-MS) analysis. Silver staining was used to visualize immunoprecipitated protein complexes prior to mass spectrometry, confirming successful enrichment of SLC25A5-associated proteins (Figure 4A). Among the candidate proteins identified by IP-MS, EIF3A was selected for further validation based on its correspondence to a prominent differential band observed in the silver staining and its high-confidence identification in the mass spectrometry analysis. Subsequent co-immunoprecipitation assays in HCT-8 and HT-29 cells supported an association between SLC25A5 and EIF3A, as endogenous SLC25A5 co-precipitated EIF3A and vice versa (Figure 4B). Furthermore, immunofluorescence staining revealed cytoplasmic co-localization of SLC25A5 (green) and EIF3A (red) (Figure 4C). Given that SLC25A5 is canonically characterized as a mitochondrial protein, we further examined its subcellular distribution. Subcellular fractionation followed by Western blot analysis showed that, although SLC25A5 is predominantly localized in mitochondria, a detectable fraction is present in the cytosolic compartment (Figure S2A,B).

We next examined whether SLC25A5 regulates EIF3A expression. RT-qPCR analysis showed no significant change in EIF3A mRNA levels upon SLC25A5 overexpression (Figure 4D). In contrast, Western blot analysis revealed a reduction in EIF3A protein levels in both HCT-8 and HT-29 cells (Figure 4E), suggesting post-transcriptional regulation. To further explore the underlying mechanism, SLC25A5-overexpressing cells were treated with pathway-specific inhibitors. The decrease in EIF3A protein levels was largely reversed by the proteasome inhibitor MG132, whereas treatment with the autophagy inhibitor chloroquine (CQ) had minimal effect (Figure 4F), indicating the involvement of the ubiquitin–proteasome system. To assess protein stability, cells were treated with the protein synthesis inhibitor cycloheximide (CHX). SLC25A5 overexpression accelerated the degradation of EIF3A, as evidenced by a shortened protein half-life in both HCT-8 (Figure 4G,H) and HT-29 (Figure 4J,K) cells. Consistently, ubiquitination assays showed increased polyubiquitination of EIF3A in SLC25A5-overexpressing cells (Figure 4I,L).

Finally, we evaluated the clinical relevance of this association in colorectal cancer tissues. Immunohistochemical staining of CRC specimens showed an inverse expression pattern between SLC25A5 and EIF3A, with samples exhibiting high SLC25A5 levels generally displaying lower EIF3A expression, and vice versa (Figure 4M). Correlation analysis further demonstrated a significant negative relationship between SLC25A5 and EIF3A protein levels in CRC samples (Figure 4N). Together, these findings support a potential role for SLC25A5 in regulating EIF3A protein stability.

### 2.5. SLC25A5 Suppresses CRC Progression in Association with EIF3A

**Figure 5:**
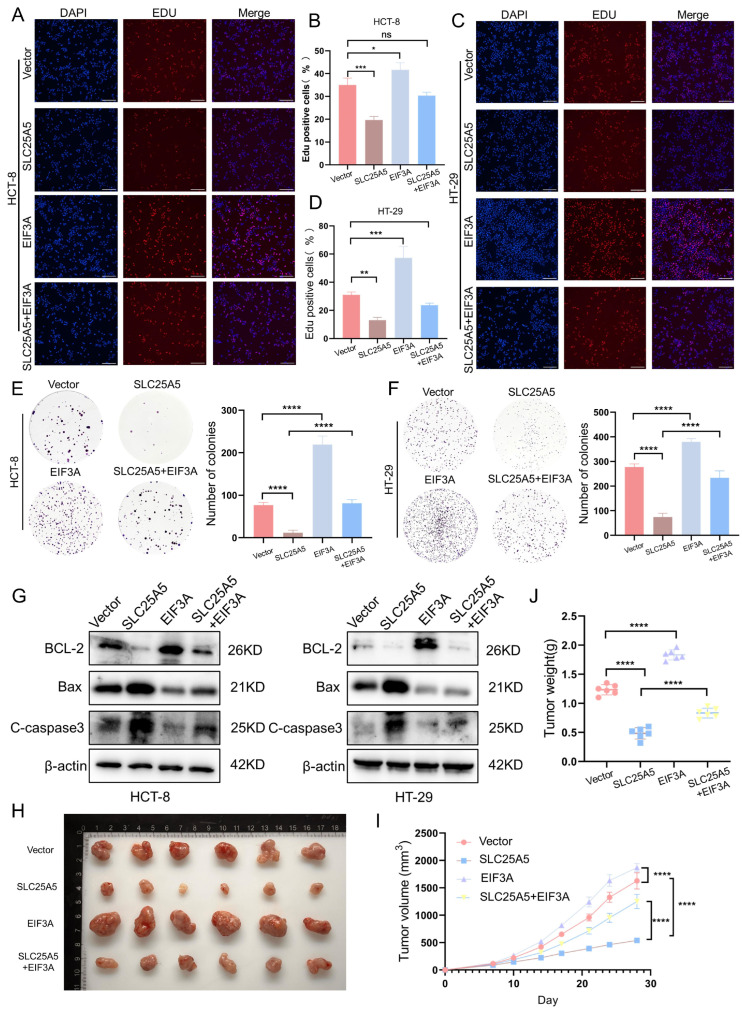
SLC25A5 Suppresses CRC Progression in Association with EIF3A: (**A**–**D**) Representative images (**A**,**C**) and quantitative analysis (**B**,**D**) of EdU incorporation assays in HCT-8 and HT-29 cells transfected with the indicated plasmids (Nuclei were stained with DAPI (blue), and proliferating cells were stained with EdU (red). Scale bars: 200 μm). (**E**,**F**) Colony formation assays of CRC cells after the indicated treatments. (**G**) Western blot analysis of Bcl-2, Bax, and cleaved-caspase3 protein levels in rescue experiments. (**H**–**J**) Representative images of xenograft tumors (**H**), tumor growth curves (**I**), and final tumor weights (**J**) from the indicated groups. * *p* < 0.05, ** *p* < 0.01, *** *p* < 0.001, **** *p* < 0.0001.

To evaluate whether the tumor-suppressive effects of SLC25A5 are functionally associated with EIF3A, we performed rescue experiments in HCT-8 and HT-29 cells. EdU incorporation assays showed that SLC25A5 overexpression reduced the proportion of proliferating cells, whereas EIF3A overexpression promoted proliferation. Notably, the inhibitory effect of SLC25A5 on DNA synthesis was partially reversed upon co-expression of EIF3A (Figure 5A–D). Consistent with these findings, colony formation assays demonstrated that SLC25A5 reduced colony numbers, an effect that was partially restored by EIF3A overexpression in both cell lines (Figure 5E,F). Furthermore, Western blot analysis indicated that SLC25A5-induced changes in apoptosis-related proteins—including increased Bax and cleaved caspase-3 and decreased Bcl-2—were attenuated upon EIF3A co-expression (Figure 5G).

To further assess the functional relevance of the SLC25A5–EIF3A axis in vivo, we established a nude mouse xenograft model (Figure 5H). Mice injected with SLC25A5-overexpressing cells exhibited reduced tumor growth, as indicated by decreased tumor volume (Figure 5I) and tumor weight (Figure 5J) compared to controls. In contrast, tumors derived from EIF3A-overexpressing cells showed increased growth. Importantly, co-expression of EIF3A partially attenuated the inhibitory effect of SLC25A5 on tumor growth, as reflected by increased tumor size and weight relative to the SLC25A5 overexpression group (Figure 5I,J). Taken together, these findings suggest that EIF3A contributes to the tumor-suppressive effects of SLC25A5 in CRC.

### 2.6. SLC25A5 Suppresses CRC Metastasis and EMT in Association with EIF3A

**Figure 6:**
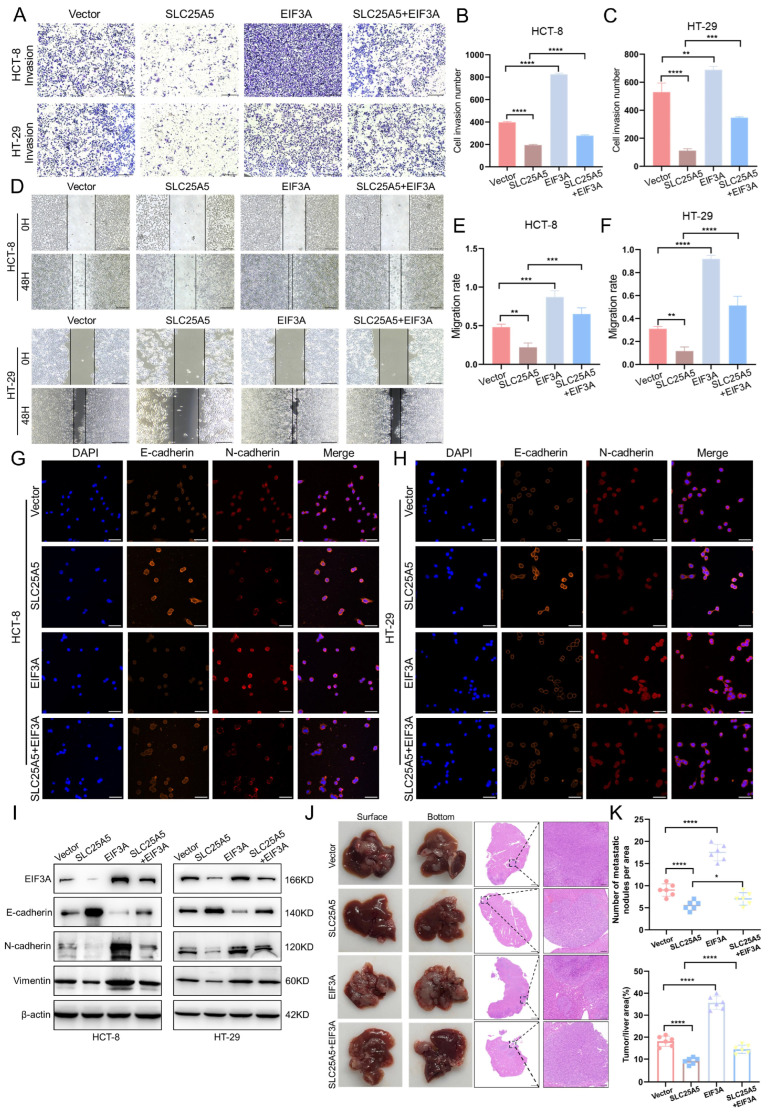
SLC25A5 Suppresses CRC Metastasis and EMT in Association with EIF3A: (**A**–**C**) Representative images (**A**) and quantification (**B**,**C**) of Transwell invasion assays in CRC cells after the indicated treatments (Scale bars: 100 μm). (**D**–**F**) Representative images (**D**) and quantitative analysis (**E**,**F**) of wound healing assays. (**G**,**H**) Immunofluorescence staining of E-cadherin (orange) and N-cadherin (red) in HCT-8 (**G**) and HT-29 (**H**) cells (Nuclei were counterstained with DAPI (blue). Scale bars: 50 μm). (**I**) Western blot analysis of EIF3A, E-cadherin, N-cadherin, and Vimentin protein levels in rescue groups. (**J**) Representative images of liver metastases and H&E staining from the indicated groups. (**K**) Statistical analysis of the number of metastatic nodules and tumor/liver area ratio. * *p* < 0.05, ** *p* < 0.01, *** *p* < 0.001, **** *p* < 0.0001.

To assess the contribution of the SLC25A5–EIF3A axis to CRC cell motility, we performed Transwell invasion and wound healing assays. Transwell assays showed that SLC25A5 overexpression reduced the number of invading cells, whereas EIF3A overexpression enhanced invasive capacity in both HCT-8 and HT-29 cell lines (Figure 6A–C). Similarly, wound healing assays demonstrated that SLC25A5 slowed wound closure, an effect that was partially attenuated by EIF3A co-expression (Figure 6D–F), suggesting that EIF3A contributes to the regulation of SLC25A5-mediated migratory phenotypes.

We next assessed the effect of SLC25A5 and EIF3A on EMT-related marker expression using immunofluorescence and Western blot analysis. Immunofluorescence staining showed that SLC25A5 overexpression increased the epithelial marker E-cadherin and decreased the mesenchymal marker N-cadherin, whereas EIF3A restoration partially reversed these changes (Figure 6G,H). Consistent with these observations, Western blot analysis showed that SLC25A5-induced changes in EMT markers—including increased E-cadherin and decreased N-cadherin and vimentin—were attenuated upon EIF3A co-expression (Figure 6I), suggesting that EIF3A is involved in SLC25A5-mediated regulation of EMT.

To further evaluate these findings in vivo, we established a liver metastasis model (Figure 6J). Mice in the SLC25A5-overexpression group developed fewer metastatic nodules and exhibited a reduced tumor-to-liver area ratio compared to controls (Figure 6K). In contrast, EIF3A overexpression was associated with increased metastatic burden. Importantly, co-expression of EIF3A partially restored metastatic potential, as reflected by increased numbers of metastatic foci and tumor area compared to the SLC25A5 overexpression group (Figure 6K), consistent with the in vitro observations. Taken together, these findings suggest that the SLC25A5–EIF3A axis is involved in the regulation of CRC metastasis and EMT.

### 2.7. SLC25A5 Suppresses CRC Progression in Association with the EIF3A–PI3K–AKT Axis

**Figure 7:**
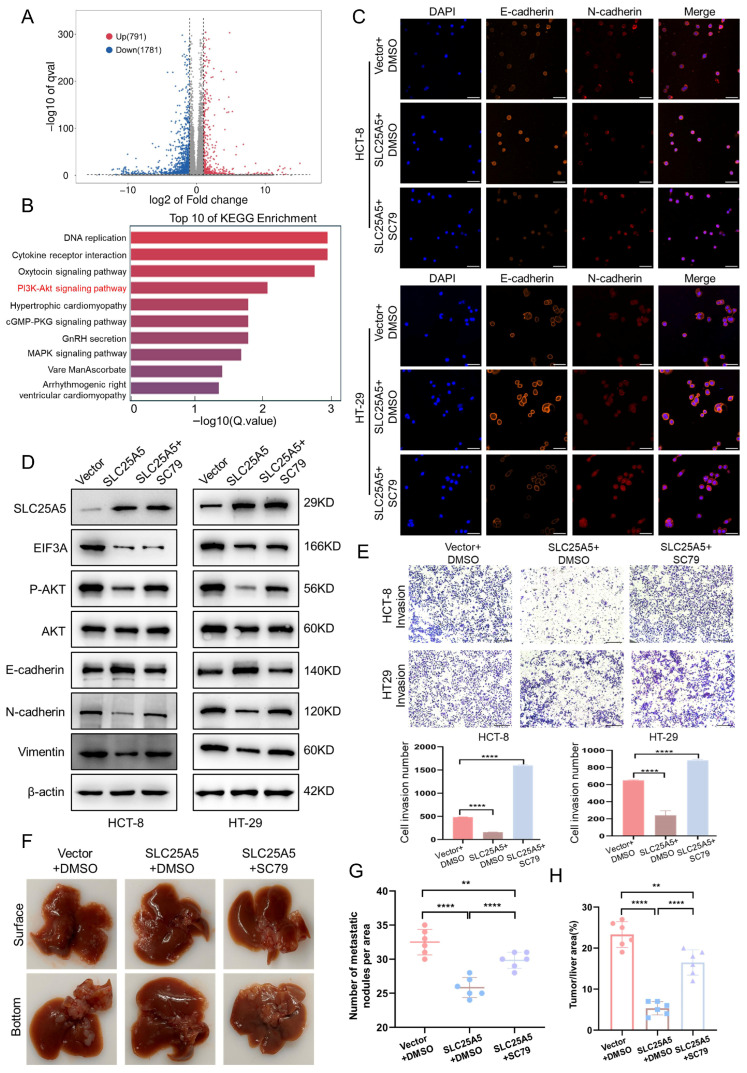
SLC25A5 Suppresses CRC Progression in Association with the EIF3A–PI3K–AKT Axis: (**A**) Volcano plot identifying differentially expressed genes (DEGs) after SLC25A5 overexpression in HCT-8 cells. (**B**) Top 10 enriched KEGG pathways based on the DEGs. (**C**) Immunofluorescence staining of E-cadherin and N-cadherin in cells treated with the AKT activator SC79 (Nuclei were counterstained with DAPI (blue). E-cadherin and N-cadherin were labeled with orange and red fluorescence, respectively. Scale bars: 50 μm). (**D**) Western blot analysis of SLC25A5, EIF3A, P-AKT, AKT, and EMT markers in the indicated groups. (**E**) Transwell invasion assays of CRC cells treated with DMSO or SC79 (Scale bars: 100 μm). (**F**–**H**) Representative liver images (**F**) and quantification of liver metastatic nodules (**G**) and tumor/liver area ratio (**H**) in the indicated groups. ** *p* < 0.01, **** *p* < 0.0001.

To explore downstream signaling pathways associated with SLC25A5, we performed RNA sequencing (RNA-seq) analysis in HCT-8 cells stably overexpressing SLC25A5 and corresponding control cells. Pearson correlation analysis confirmed high reproducibility among biological replicates (Figure S3A), and principal component analysis (PCA) showed clear separation between groups, with PC1 accounting for 36.04% of the variance (Figure S3B). Differential expression analysis identified 791 upregulated and 1781 downregulated genes (|log2 fold change| ≥ 1, adjusted *p* < 0.05) following SLC25A5 overexpression (Figure 7A). KEGG pathway enrichment analysis indicated that the PI3K–AKT signaling pathway was among the most significantly enriched pathways (Figure 7B). Gene set enrichment analysis (GSEA) further showed that PI3K–AKT signaling was downregulated in the SLC25A5-overexpression group (Figure S3C).

To further examine the relationship between SLC25A5, EIF3A, and AKT signaling, we analyzed protein expression by Western blot. SLC25A5 overexpression reduced phosphorylated AKT (p-AKT) levels without affecting total AKT. Notably, EIF3A co-expression partially restored p-AKT levels, suggesting that EIF3A contributes to the regulation of AKT signaling in this context (Figure S3D). Consistent with these observations, reduced p-AKT levels were also observed in SLC25A5-overexpressing HCT-8 and HT-29 cells (Figure 7D). Together, these findings suggest that SLC25A5 is associated with suppression of PI3K–AKT signaling.

We next examined whether PI3K–AKT signaling contributes to the phenotypic effects of SLC25A5. Treatment with the AKT activator SC79 restored p-AKT levels in SLC25A5-overexpressing cells (Figure 7D). Notably, SLC25A5-induced changes in EMT markers were partially reversed following SC79 treatment, including decreased E-cadherin and increased mesenchymal marker expression (Figure 7D). Immunofluorescence analysis further supported these findings, showing that SC79 attenuated the SLC25A5-mediated changes in E-cadherin and N-cadherin expression (Figure 7C). In addition, the reduced invasive capacity of CRC cells induced by SLC25A5 was partially restored upon SC79 treatment (Figure 7E).

To further assess the relevance of PI3K–AKT signaling in vivo, we established a liver metastasis model (Figure 7F). SLC25A5 overexpression reduced metastatic burden, as indicated by fewer metastatic nodules and a lower tumor-to-liver area ratio. Importantly, SC79 treatment partially attenuated these effects, resulting in increased metastatic burden compared to the SLC25A5 overexpression group (Figure 7G,H). Taken together, these findings suggest that the PI3K–AKT pathway is involved in SLC25A5-mediated regulation of CRC metastasis and EMT.

## 3. Discussion

In this study, we provide evidence supporting SLC25A5 as a tumor-suppressive factor in colorectal cancer and propose a regulatory axis involving SLC25A5, EIF3A, and the PI3K/AKT signaling pathway. By integrating clinical cohort analyses with functional assays, we show that SLC25A5 deficiency promotes CRC proliferation, epithelial–mesenchymal transition, and systemic metastasis. These findings suggest a previously underappreciated, non-canonical role for this mitochondrial carrier protein in modulating cytoplasmic oncogenic signaling.

SLC25A5 has traditionally been characterized as a mitochondrial ADP/ATP exchanger, with its role in malignancy reported as context-dependent [20,21,22]. Unlike certain cancers where SLC25A5 facilitates glycolytic flux and tumor growth [23,24,25], our multi-cohort analyses reveal a contrasting pattern in CRC. In this context, SLC25A5 expression is significantly downregulated and positively correlates with patient survival. Single-cell transcriptomic profiling further indicates that SLC25A5 expression is primarily enriched within malignant epithelial populations, supporting a cell-intrinsic tumor-suppressive function. Together, these observations suggest that the biological impact of SLC25A5 in CRC may be tissue-specific and extend beyond its canonical metabolic activity.

A central aspect of our study is the potential extra-mitochondrial localization and non-canonical activity of SLC25A5. While SLC25A5 is classically defined as a mitochondrial inner membrane protein [26], our data suggest the presence of a cytoplasmic pool in CRC cells. Confocal microscopy revealed spatial co-localization between SLC25A5 and EIF3A. Consistently, subcellular fractionation assays detected SLC25A5 in the cytosolic fraction, in the absence of mitochondrial markers. From a structural perspective, the solvent-exposed hydrophilic loops of SLC25A5 may provide a potential interface for interaction with cytoplasmic proteins [27,28]. This observation is consistent with the “protein moonlighting” concept, in which mitochondrial proteins exhibit context-dependent localization or functions beyond their canonical roles [29,30,31]. However, the precise subcellular distribution and trafficking mechanism of SLC25A5 remain to be further elucidated.

Mechanistically, our data suggest that SLC25A5 facilitates the destabilization of EIF3A through the ubiquitin–proteasome system. Co-immunoprecipitation and co-localization analyses support a physical and functional association between these two proteins; however, the precise biochemical nature of this interaction—specifically whether it represents direct binding or is mediated by a multi-protein complex—remains to be determined. Future studies incorporating in vitro binding assays and domain-mapping approaches will be required to clarify this interaction. Notably, the accelerated turnover of EIF3A upon SLC25A5 overexpression, together with its rescue by MG132, supports a role for SLC25A5 in regulating EIF3A protein stability.

Furthermore, our findings indicate that SLC25A5-mediated reduction in EIF3A is associated with attenuation of PI3K/AKT signaling. Pharmacological reactivation of AKT using SC79 partially rescued the EMT and invasive phenotypes, supporting the functional involvement of this pathway in SLC25A5-mediated tumor suppression. However, the precise molecular link between EIF3A and AKT phosphorylation remains to be fully elucidated and represents a limitation of the current study. Given that EIF3A is a core component of the translation initiation machinery [32,33], it is plausible that key regulators of the PI3K/AKT pathway may serve as downstream translational targets. In this context, stabilization of EIF3A could enhance the translation of these signaling mediators, which may contribute to sustained AKT activation. Nevertheless, the lack of direct translatome profiling, such as ribosome profiling (Ribo-seq), precludes definitive identification of these targets at present.

We acknowledge that, although our study proposes a regulatory axis involving SLC25A5 and EIF3A, several mechanistic questions remain unresolved. In particular, the identity of the E3 ligases potentially involved in EIF3A ubiquitination downstream of SLC25A5 requires further investigation. Therefore, our findings should be interpreted as an initial step toward understanding the non-canonical roles of mitochondrial carrier proteins. Rather than providing a complete biochemical characterization of this process, our study highlights the potential for SLC25A5 to participate in cytoplasmic protein homeostasis and to functionally connect organelle-associated processes with oncogenic signaling pathways. Targeting the SLC25A5–EIF3A axis may offer a potential therapeutic avenue for metastatic CRC, although this possibility requires further validation.

In summary, we propose a tumor-suppressive axis in CRC in which loss of SLC25A5 is associated with EIF3A stabilization and sustained PI3K/AKT signaling, thereby promoting EMT and metastatic progression. While the detailed molecular mechanisms underlying this axis remain to be fully defined, our findings provide a conceptual framework for exploring how mitochondrial carrier proteins may contribute to signaling regulation beyond their canonical metabolic functions.

## 4. Materials and Methods

### 4.1. Patient Cohorts and Clinical Specimens

Public Bulk Transcriptomic Cohorts: Transcriptome profiling data and corresponding clinical information were obtained from The Cancer Genome Atlas (TCGA-COAD) database. Two independent validation datasets (GSE32323 and GSE39582) were downloaded from the Gene Expression Omnibus (GEO) database. These cohorts were utilized for differential expression analysis between tumor and adjacent normal tissues, as well as for Kaplan–Meier survival analysis.

Public Single-Cell RNA Sequencing (scRNA-seq) Dataset: The scRNA-seq dataset CRC-EMTAB8107 was retrieved to evaluate the specific cellular distribution of SLC25A5 within the complex CRC tumor microenvironment.

Local Clinical Specimens: Primary CRC tissues and matched adjacent normal tissues were retrieved from the tissue archive at the School of Medicine of Tongji University. For experimental validation, snap-frozen tissues were subjected to RT-qPCR (n = 20 pairs) and Western blot analysis (n = 10 pairs). Furthermore, paraformaldehyde-fixed, paraffin-embedded tissues from 20 patient pairs were utilized for immunohistochemistry (IHC). All procedures were approved by the Institutional Ethics Committee of Tongji University (No. SHSY-IEC-5.0/24K84/P01), and written informed consent was obtained from all participating patients.

### 4.2. RNA Extraction, Sequencing, and Bioinformatics Analysis

Total RNA was extracted from both local clinical specimens and cultured cell lines using TRIzol reagent (Invitrogen, Carlsbad, CA, USA). Subsequently, complementary DNA (cDNA) synthesis was performed utilizing the PrimeScript RT Reagent Kit (TaKaRa, Kyoto, Japan). Quantitative real-time PCR (qRT-PCR) was conducted with SYBR Green Master Mix on an ABI 7500 Real-Time PCR System. All primer sequences utilized for amplification are provided in Supplementary Table S1. The relative expression levels of target genes were calculated via the 2^−ΔΔCT^ method, with GAPDH serving as the internal reference control.

Public Bulk RNA-seq Bioinformatics Analysis. These cohorts were utilized for differential expression analysis between tumor and adjacent normal tissues, as well as for Kaplan–Meier survival analysis. Specifically, the expression profiles of SLC25A5 in both the TCGA and GEO databases were systematically evaluated using the R statistical programming environment (version 4.3.1). For the GEO dataset, the limma package (version 3.58.1) was employed for data normalization and differential analysis. For the TCGA cohort, the DESeq2 package (version 1.40.2) was utilized to process the RNA-sequencing data, and the ggplot2 package was used for data visualization. Genes with |log_2_ fold change| ≥ 1 and adjusted *p* < 0.05 were considered significantly differentially expressed.

To assess the prognostic value of SLC25A5, Kaplan–Meier survival curves for the TCGA cohort were generated using the TIMER 3.0 web server (http://timer.cistrome.org/ (accessed on 15 March 2025)). Furthermore, survival analysis for the GEO dataset (GSE39582) was conducted via the Kaplan–Meier Plotter (https://kmplot.com/analysis/ (accessed on 15 March 2025)), where patients were stratified into high and low expression groups based on the ‘best cutoff’ value (a threshold determined by the maximally selected rank statistics to provide the lowest *p*-value) to ensure the most significant separation of survival outcomes.

Single-Cell RNA-seq Bioinformatics Analysis. To evaluate the specific cellular distribution of SLC25A5 within the complex colorectal cancer (CRC) tumor microenvironment, the scRNA-seq dataset E-MTAB-8107 was analyzed utilizing the TISCH2 (Tumor Immune Single-cell Hub 2) web platform (http://tisch.comp-genomics.org/ (accessed on 15 March 2025)).

In-house Bulk RNA Sequencing and Alignment. For the in-house constructed HCT-8 cell lines, RNA libraries were prepared and sequenced on an Illumina NovaSeq platform. Raw reads underwent quality control and were aligned to the human reference genome (GRCh38.p14, Ensembl release 109) using HISAT2 (version 2.2.1). Gene expression quantification and differential expression analysis were performed using the DESeq2 package in R software (version 4.3.1). Genes with |log_2_ fold change| ≥ 1 and adjusted *p* < 0.05 were considered significantly differentially expressed. Downstream KEGG pathway enrichment analysis was conducted using the clusterProfiler package (version 4.8.1).

### 4.3. Western Blot Analysis

Total proteins were extracted from the cultured cells and tissue samples using RIPA lysis buffer (Beyotime, Shanghai, China) supplemented with protease and phosphatase inhibitor cocktails (Roche, Basel, Switzerland). Protein concentrations were determined using a BCA Protein Assay Kit (Thermo Fisher Scientific, Waltham, MA, USA). Equal amounts of protein samples (30 μg/lane) were separated by 10% sodium dodecyl sulfate-polyacrylamide gel electrophoresis (SDS-PAGE) and subsequently transferred onto polyvinylidene fluoride (PVDF) membranes (Millipore, St. Louis, MA, USA). After blocking with 5% non-fat milk in Tris-buffered saline containing 0.1% Tween 20 (TBST) for 1 h at room temperature, the membranes were incubated with specific primary antibodies overnight at 4 °C. Following three washes with TBST, the membranes were incubated with horseradish peroxidase (HRP)-conjugated secondary antibodies for 1 h at room temperature. The protein bands were visualized using an enhanced chemiluminescence (ECL) detection system (Bio-Rad, Hercules, CA, USA), and the relative band intensities were quantified using ImageJ software (version 1.54f, NIH, Bethesda, MD, USA). β-actin was used as an internal loading control to normalize protein expression. Detailed information regarding all primary and secondary antibodies used in this study is provided in Supplementary Table S2.

### 4.4. Immunohistochemistry (IHC) Staining

The formalin-fixed, paraffin-embedded (FFPE) tissue sections (4 μm thick) were deparaffinized in xylene and rehydrated through a graded series of ethanol. For antigen retrieval, the sections were treated with citrate buffer (pH 6.0) or EDTA buffer (pH 9.0) using a pressure cooker for 15 min. Endogenous peroxidase activity was blocked with 3% hydrogen peroxide for 15 min at room temperature. After washing with PBS, the sections were incubated with 5% normal goat serum for 30 min to reduce non-specific binding, followed by incubation with primary antibodies overnight at 4 °C. On the following day, the sections were incubated with HRP-conjugated secondary antibodies for 1 h at room temperature. The protein expression was visualized using 3,3′-diaminobenzidine (DAB) substrate, and the sections were counterstained with hematoxylin. The IHC results were independently evaluated by two experienced researchers in a blinded manner. The protein expression level was quantified using the IHC score (H-score) system, which was calculated based on the staining intensity (0, negative; 1, weak; 2, moderate; 3, strong) and the percentage of positive cells (0, <5%; 1, 5–25%; 2, 26–50%; 3, 51–75%; 4, >75%). The final IHC score, ranging from 0 to 12, was determined by multiplying the intensity score by the percentage score.

### 4.5. Cell Culture and Stable Line Construction

Human CRC cell lines HCT-8 and HT-29 were obtained from ATCC and cultured in RPMI-1640 or McCoy’s 5A medium supplemented with 10% fetal bovine serum (FBS) and 1% penicillin/streptomycin at 37 °C in a humidified incubator containing 5% CO_2_. Stable overexpression of SLC25A5 or EIF3A was achieved using lentiviral vectors (pLenti-SLC25A5, pLenti-EIF3A, or empty vector). Cells were transduced in the presence of polybrene (8 μg/mL) and selected with puromycin (2 μg/mL) for 14 days to establish stable cell lines.

### 4.6. In Vitro Functional Assays

#### 4.6.1. Proliferation (CCK-8, EdU) and Colony Formation

For cell proliferation analysis, cells were seeded in 96-well plates at a density of 2000 cells/well and cultured for up to 96 h. Cell viability was assessed daily using the Cell Counting Kit-8 (CCK-8) according to the manufacturer’s instructions. Additionally, proliferating cells were visually detected using an EdU cell proliferation kit (Beyotime, Shanghai, China) following the standard protocol provided by the manufacturer. For colony formation assays, cells were seeded into 6-well plates at a density of 1000 cells/well and maintained in culture for 14 days. The resulting colonies were fixed with methanol, stained with 0.1% crystal violet, and subsequently counted.

#### 4.6.2. Apoptosis Analysis

Cell apoptosis was assessed using an APC-PI Apoptosis Detection Kit (Beyotime, Shanghai, China) according to the manufacturer’s instructions. Stained cells were analyzed by flow cytometry, and the apoptotic cell populations were quantified using FlowJo software (version 10.8.1).

#### 4.6.3. Migration and Invasion (Wound Healing and Transwell)

For wound healing assays, a linear scratch was generated in confluent cell monolayers using a sterile pipette tip. Images were captured at 0, 24, and 48 h to evaluate migratory capacity. The wound closure area was quantified using ImageJ software (version 1.54f, National Institutes of Health, Bethesda, MD, USA), and the migration rate was calculated using the following formula: (Area at 0 h − Area at t h)/Area at 0 h. For Transwell assays, cells suspended in serum-free medium were seeded in the upper chambers. For invasion assays, the upper chambers were pre-coated with Matrigel. Medium containing 20% FBS was added to the lower chamber as a chemoattractant. After 24–48 h of incubation, the migrated or invaded cells on the lower surface of the membrane were fixed and stained. To quantitatively assess migration and invasion capacities, cells were counted in 5 randomly selected fields per well under a light microscope.

### 4.7. Co-Immunoprecipitation (Co-IP) and Mass Spectrometry (MS)

To identify the potential interacting proteins of SLC25A5, Co-IP assays were performed in HCT8 and HT29 cells. Briefly, total proteins were extracted using a mild IP lysis buffer (Thermo Fisher Scientific, USA) containing protease and phosphatase inhibitors. The cell lysates were incubated with a specific anti-SLC25A5 antibody or a control IgG overnight at 4 °C with gentle rotation, followed by the addition of Protein A/G magnetic beads for an additional 2 h. The beads were then washed, and the immunoprecipitated complexes were eluted and separated by SDS-PAGE. For MS analysis, the protein bands were excised and subjected to in-gel trypsin digestion. The resulting peptides were analyzed by liquid chromatography-tandem mass spectrometry (LC-MS/MS). Protein identification was performed by searching the raw data against the UniProt human database. Detailed information regarding the antibodies used is provided in Supplementary Table S1.

### 4.8. Protein Stability and Ubiquitination Assays

To assess EIF3A protein stability, cells were treated with the protein synthesis inhibitor cycloheximide (CHX, 100 μg/mL) and harvested at 0, 2, 4, 8, 12, and 24 h. To determine the degradation pathway, cells were treated with the proteasome inhibitor MG132 (10 μM) or the autophagy inhibitor chloroquine (CQ, 50 μM) for 6 h before protein extraction. For ubiquitination assays, cells were pre-treated with MG132 (10 μM) for 6 h. Cells were then lysed under denaturing conditions in RIPA buffer supplemented with 1% SDS and N-ethylmaleimide (NEM) to inhibit deubiquitinating enzymes. Lysates were diluted to reduce SDS concentration before immunoprecipitation with an anti-EIF3A antibody. Precipitated proteins were subjected to Western blot analysis using an anti-ubiquitin antibody to detect poly-ubiquitinated EIF3A.

### 4.9. Immunofluorescence (IF) Staining and Co-Localization Analysis

For immunofluorescence analysis, CRC cells (HCT-8 and HT-29) were seeded on glass coverslips in 24-well plates and cultured until they reached approximately 60–70% confluence. The cells were fixed with 4% paraformaldehyde for 15 min at room temperature, followed by permeabilization with 0.1% Triton X-100 in PBS for 10 min. After blocking with 5% bovine serum albumin (BSA) in PBS for 1 h to minimize non-specific binding, the coverslips were incubated overnight at 4 °C with primary antibodies against SLC25A5 (1:100; Abclonal, Woburn, MA, USA, A23411), EIF3A (1:400; Cell Signaling Technology, Danvers, MA, USA, 3411), E-cadherin (1:1600; Cell Signaling Technology, 9782), or N-cadherin (1:400; Cell Signaling Technology, 14215). On the following day, the cells were washed three times with PBS and then incubated with corresponding Alexa Fluor-conjugated secondary antibodies (e.g., Alexa Fluor 488 or 594) for 1 h at room temperature in the dark. Nuclei were counterstained with DAPI (Beyotime, Shanghai, China) for 5 min to visualize the DNA. For co-localization studies of SLC25A5 and EIF3A, double-staining was performed using the respective primary and secondary antibodies. Finally, the coverslips were mounted onto glass slides using an anti-fade mounting medium. Images were captured using a confocal laser scanning microscope (or fluorescence microscope) to evaluate the expression patterns of EMT markers and the spatial distribution of SLC25A5 and EIF3.

### 4.10. In Vivo Xenograft and Metastasis Models

All animal procedures were performed in accordance with the Guide for the Care and Use of Laboratory Animals and were approved by the Institutional Animal Care and Use Committee of Tongji University (No. SHDSYY-2021-3932). Mice were randomly assigned to experimental groups. For xenograft assays, 5 × 10^6^ stable HCT-8 cells were subcutaneously injected into the flanks of 4-week-old male nude mice (n = 6 per group). Tumor volume was measured every 4 days using the formula:

Volume = 0.5 × Length × Width^2^.

For liver metastasis models, cells were injected into the spleen of nude mice. In rescue experiments, SC79 (20 mg/kg) or DMSO was administered intraperitoneally. After 4–6 weeks, mice were sacrificed, and livers were harvested for metastatic nodule counting and H&E staining.

### 4.11. Statistical Analysis

All quantitative data are presented as mean ± standard deviation (SD) from at least three independent experiments unless otherwise specified. Statistical analyses were performed using GraphPad Prism (version 10.1). Comparisons between two groups were conducted using a two-tailed unpaired Student’s *t*-test. Comparisons among multiple groups were performed using one-way ANOVA followed by Tukey’s post hoc test. Kaplan–Meier survival curves were compared using the log-rank test. Pearson correlation analysis was used to assess the relationship between SLC25A5 and EIF3A expression levels. A *p* value < 0.05 was considered statistically significant.

## Data Availability

The data that support the findings of this study are available from the corresponding author upon reasonable request.

## Supplementary Materials

The following supporting information can be downloaded at: https://www.mdpi.com/article/10.3390/ijms27104334/s1.

## Abbreviations

The following abbreviations are used in this manuscript:

ANT2	Adenine nucleotide translocator 2
CHX	Cycloheximide
Co-IP	Co-immunoprecipitation
CQ	Chloroquine
CRC	Colorectal cancer
DEG	Differentially expressed gene
EIF3A	Eukaryotic translation initiation factor 3 subunit A
EMT	Epithelial–mesenchymal transition
GEO	Gene Expression Omnibus
KEGG	Kyoto Encyclopedia of Genes and Genomes
NAT	Normal adjacent tissue
P-AKT	Phosphorylated AKT
PI3K/AKT	Phosphoinositide 3-kinase/protein kinase B signaling pathway
SLC25A5	Solute carrier family 25 member 5
UMAP	Uniform manifold approximation and projection
UPS	Ubiquitin-proteasome system
