# Supplementary material for: SLC25A5 Suppresses Colorectal Cancer Growth and Metastasis Through Regulation of the EIF3A/PI3K/AKT Axis
Source: Int J Mol Sci. 2026 May 13;27(10):4334. doi: 10.3390/ijms27104334 (PMC13207500; doi:10.3390/ijms27104334)
Supplement: Supplementary file 1 [file ijms-27-04334-s001.zip › ijms-4253356-supplementary.pdf]

Supplementary Materials

SLC25A5 Suppresses Colorectal Cancer Growth and Metastasis through Regulation of the EIF3A/PI3K/AKT Axis

Ke Ying [1#], Xiang Zhao [1#], Zhuo Wu [1], Chi Huang [1], Qian Wu [1], Zhongchen Liu [1,*]

Table S1. Primer sequences used in this study.

Gene	Primer	Sequence (5'-3')
SLC25A5	Forward primer	GCTGCTGGTGTTTGCTGTTG
	Reverse primer	CAGGATGACAGCAGCAGGAT
EIF3A	Forward primer	TGGACCTGAAGGAGGAGATG
	Reverse primer	CTGCTTGTTGATGGTGGTGA
β-actin	Forward primer	CATGTACGTTGCTATCCAGGC
	Reverse primer	CTCCTTAATGTCACGCACGAT

Table S2. Antibodies used in this study.

Antibody	Supplier	Catalog number	Application	Dilution
anti-huaman-SLC25A5	Cell Signaling Technology	14671	IB, IP	1:1000,1:50
anti-huaman-SLC25A5	Bioss	bs-9567R	IHC	1:300
anti-huaman-SLC25A5	Abclonal	A23411	IF	1:100
anti-huaman-BCL-2	Proteintech	12789-1-AP	IB	1:5000
anti-huaman-Bax	Proteintech	50599-2-Ig	IB	1:20000
anti-huaman-Cleaved Caspase 3	Proteintech	25128-1-AP	IB	1:1000
anti-human-E-Cadherin	Cell Signaling Technology	9782	IB, IF	1:1000,1:1600
anti-human-Vimentin	Cell Signaling Technology	9782	IB	1:1000
anti-human-N-Cadherin	Cell Signaling Technology	9782	IB	1:1000
anti-human-N-Cadherin	Cell Signaling Technology	14215	IF	1:400
anti-human-EIF3A	Cell Signaling Technology	3411	IB, IP, IF, IHC	1:1000,1:100, 1:400,1:300
anti-human-DDDDK-Tag	Abclonal	AE092	IB, IF, IP	1:1000, 1:100, 1:100
anti-human-HA-Tag	Abclonal	AE105	IB, IF, IP	1:1000, 1:100, 1:100
anti-human-Ubiquitin	Abclonal	A19686	IB	1:2000
anti-human-β-actin	Abclonal	AC026	IB	1:10000
anti-human-AKT	Selleck	F0004	IB	1:1000
anti-human-AKT (phospho S127)	Proteintech	66444-1-Ig	IB	1:2000

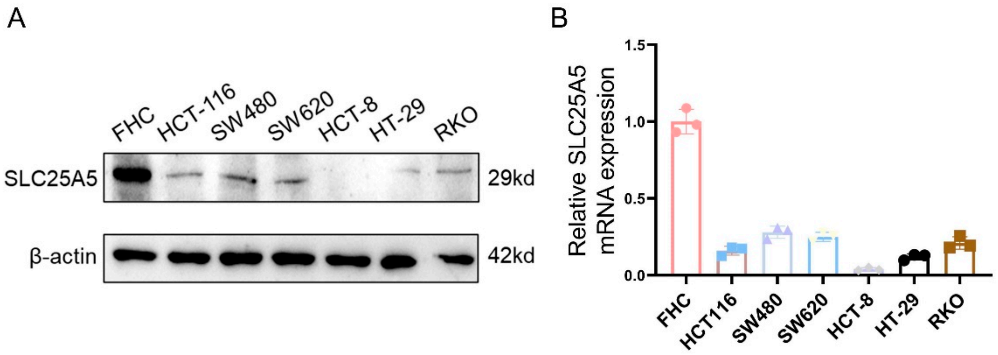

Fig S1 Endogenous expression of SLC25A5 in CRC cell lines. (A) Representative western blot analysis of endogenous SLC25A5 protein levels across a panel of human colorectal cancer (CRC) cell lines (HCT-116, SW480, SW620, HCT-8, HT-29, and RKO) compared to the normal human colon epithelial cell line (FHC). (B) Quantitative RT-PCR analysis of SLC25A5 mRNA expression in the indicated cell lines. Data are presented as mean ± SD from three independent experiments.

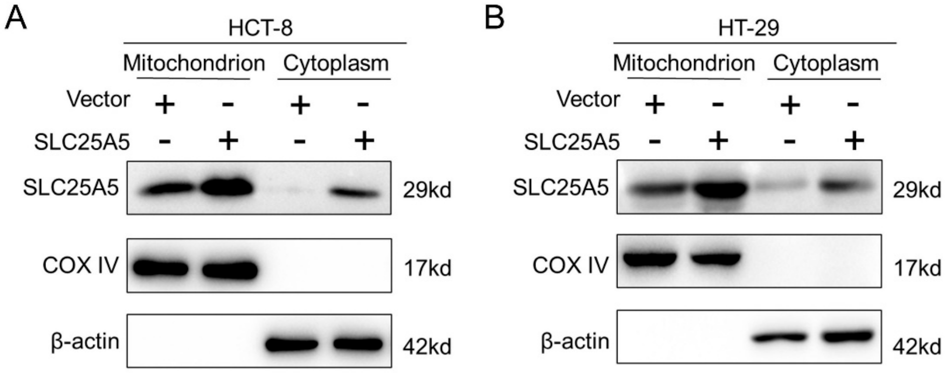

Fig S2 Subcellular localization of SLC25A5 in CRC cells. (A-B) Subcellular fractionation analysis of SLC25A5 protein levels in HCT-8 (A) and HT-29 (B) cells. COX IV was used as a marker for the mitochondrial fraction, and β-actin served as a marker for the cytoplasmic fraction. SLC25A5 was detected in both the mitochondrial and cytoplasmic compartments across both cell lines, with no evidence of cross-contamination as validated by the compartment-specific markers.

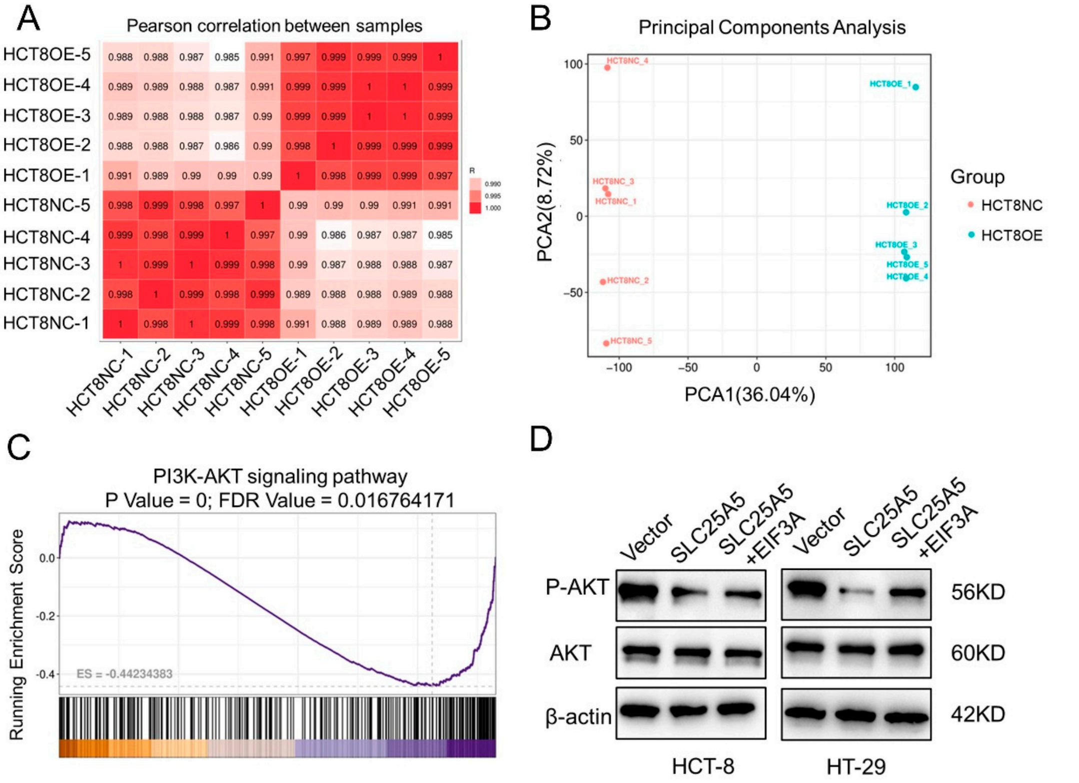

Fig S3 SLC25A5 overexpression modulates the PI3K-AKT signaling pathway in colorectal cancer cells. (A) Pearson correlation heatmap illustrating the high degree of reproducibility between biological replicates within the control (HCT8NC) and SLC25A5-overexpressing (HCT8OE) groups. **(B)** Principal Component Analysis (PCA) showing distinct clustering and clear separation between the HCT8NC (red) and HCT8OE (teal) samples. **(C)** Gene Set Enrichment Analysis (GSEA) plot for the PI3K-AKT signaling pathway. The negative enrichment score indicates a significant downregulation or modulation of this pathway following the manipulation of SLC25A5. **(D)** Western blot analysis of the SLC25A5/EIF3A/AKT axis in HCT-8 and HT-29 cells. The results show that SLC25A5 overexpression markedly suppresses P-AKT protein levels without affecting total AKT. Notably, the co-overexpression of EIF3A effectively rescued the SLC25A5-induced downregulation of P-AKT.
